# Outcomes of people living with HIV after hospital discharge: a systematic review and meta-analysis

**DOI:** 10.1016/S2352-3018(21)00329-5

**Published:** 2022-03-01

**Authors:** Nathan Ford, Gabriela Patten, Ajay Rangaraj, Mary-Ann Davies, Graeme Meintjes, Tom Ellman

**Affiliations:** a Department of Global HIV, Hepatitis and Sexually Transmitted Infections Programmes, WHO, Geneva, Switzerland; b Centre for Infectious Disease Epidemiology and Research, School of Public Health and Family Medicine, University of Cape Town, Cape Town, South Africa; c Department of Medicine, Faculty of Health Sciences, University of Cape Town, Cape Town, South Africa; d Wellcome Centre for Infectious Diseases Research in Africa, Institute of Infectious Disease and Molecular Medicine, University of Cape Town, Cape Town, South Africa; e Southern Africa Medical Unit, Médecins Sans Frontières, Cape Town, South Africa

## Abstract

**Background:**

The identification and appropriate management of people with advanced HIV disease is a key component in the HIV response. People with HIV who are hospitalised are at a higher risk of death, a risk that might persist after discharge. The aims of this study were to estimate the frequency of negative post-discharge outcomes, and to determine risk factors for such outcomes in people with HIV.

**Methods:**

Using a broad search strategy combining terms for hospital discharge and HIV infection, we searched MEDLINE via PubMed and Embase from Jan 1, 2003 to Nov 30, 2021 to identify studies reporting outcomes among people with HIV following discharge from hospital. We estimated pooled proportions of readmissions and deaths after hospital discharge using random-effects models. We also did subgroup analyses by setting, region, duration of follow-up, and advanced HIV status at admission, and sensitivity analyses to assess heterogeneity.

**Findings:**

We obtained data from 29 cohorts, which reported outcomes of people living with HIV after hospital discharge in 92 781 patients. The pooled proportion of patients readmitted to hospital after discharge was 18·8% (95% CI 15·3–22·3) and 14·1% (10·8–17·3) died post-discharge. In sensitivity analyses, no differences were identified in the proportion of patients who were readmitted or died when comparing studies published before 2016 with those published after 2016. Post-discharge mortality was higher in studies from Africa (23·1% [16·5–29·7]) compared with the USA (7·5% [4·4–10·6]). For studies that reported both post-discharge mortality and readmission, the pooled proportion of patients who had this composite adverse outcome was 31·7% (23·9–39·5). Heterogeneity was moderate, and largely explained by patient status and linkage to care. Reported risk factors for readmission included low CD4 cell count at admission, longer length of stay, discharge against medical advice, and not linking to care following discharge; inpatient treatment with antiretroviral therapy (ART) during hospitalisation was protective of post-discharge mortality.

**Interpretation:**

More than a quarter of patients with HIV had an adverse outcome after hospital discharge with no evidence of improvement in the past 15 years. This systematic review highlights the importance of ensuring post-discharge referral and appropriate management, including ART, to reduce mortality and readmission to hospital among this group of high-risk patients.

**Funding:**

Bill & Melinda Gates Foundation.

**Translations:**

For the French and Spanish translations of the abstract see Supplementary Materials section.

## Introduction

The identification and appropriate management of people with advanced HIV disease is a key component in the HIV response to further reduce HIV-related mortality. Hospitalisations from complications associated with HIV infection, including coinfections associated with advanced HIV such as tuberculosis, cryptococcal meningitis, or severe bacterial infections remain substantial. A 2015 review found that HIV-related infections and bacterial infections are leading causes of hospital admission among people living with HIV.^1^ Low CD4 cell count and low antiretroviral coverage at admission are major contributors to this disease profile and associated mortality.^1^, ^2^

People with HIV who are hospitalised are at a higher risk of death,^2^, ^3^, ^4^, ^5^, ^6^, ^7^ and this risk might persist after discharge.^5^, ^8^ A systematic review of post-discharge mortality among general paediatric admissions in low-income settings found that post-discharge mortality rates often exceeded in-hospital mortality rates.^9^ A study from South Africa found that even with widespread access to antiretroviral therapy (ART), the majority of inpatient deaths were among patients with HIV.^10^ HIV is also commonly associated with readmission following discharge.^11^ Another study from South Africa reported that 6 months after discharge, half of patients had been readmitted at least once and a quarter had died.^5^ Several studies have identified factors associated with poor post-discharge outcomes among people living with HIV, including low CD4 cell count,^12^ lack of ART,^13^ and discharge against medical advice.^14^

The aims of this systematic review and meta-analysis were to assess post-discharge outcomes of people living with HIV and to summarise risk factors associated with poor outcomes.

**Research in context**

**Evidence before this study**

There has been a renewed focus on advanced HIV disease in the past 5 years, with a need to improve outcomes to reduce HIV-related mortality. Individuals with advanced HIV disease (CD4 count <200 cells per μL) are at an increased risk of mortality from tuberculosis, cryptococcal meningitis, severe bacterial infections, and several other infectious diseases due to severely compromised immune function. In 2017, WHO recommended a package of care to be offered to individuals diagnosed with advanced HIV disease. Interventions in the package of care are directed at preventing, diagnosing, and treating the major causes of HIV-associated mortality and have been included in guidelines in many countries worldwide. Mortality among patients with HIV in hospital has been summarised, but the outcomes of patients after hospital discharge are less well understood.

**Added value of this study**

This systematic review provides summary estimates of the high rates of death or readmission following hospital discharge among people with HIV, and identified several studies reporting on risk factors that affect post-discharge outcomes. The proportion of patients with adverse outcomes has remained unchanged since 2016 and was similar in high-income and low-income settings. Post-discharge mortality and readmissions were associated with advanced HIV disease, lack of antiretroviral therapy, and discharge against medical advice among patients who were admitted to hospital.

**Implications of all the available evidence**

Ensuring adequate care for individuals with advanced HIV disease continues to be a significant challenge in many high burden settings. These findings emphasise the need for continued efforts to ensure that all people with HIV are taking antiretroviral therapy and that better support is provided for post-discharge referral and appropriate post-discharge linkage and management, particularly for individuals with low CD4 cell counts at discharge. It is important that programmes are able to collect data and report outcomes for people with HIV who are admitted to hospital to better inform progress towards global targets. Approaches are needed to identify individuals at the highest risk of poor outcomes and to provide adapted support.

## Methods

### Search strategy and selection criteria

This systematic review and meta-analysis adhered to the Preferred Reporting Items for Systematic Reviews and Meta-Analysis (PRISMA) statement.^15^ The study protocol is available in appendix 3 (pp 5–8).

To be included, studies had to report outcomes among people living with HIV following discharge from hospital. We were specifically interested in studies that reported outcomes following hospital discharge after Jan 1, 2003, when ART scale-up in low-income countries began. Studies had to report outcomes for at least 20 patients following discharge. We searched MEDLINE via PubMed and Embase from Jan 1, 2003 to Nov 30, 2021, using a highly sensitive search strategy that combined terms for HIV infection, hospital admission or readmission, and discharge, without language, geographical, or age restrictions. Full search strategies are available in the protocol (appendix 3 pp 5–8). We also screened references of review articles and all included full-text articles, and all articles that were included in a previous systematic review of outcomes after hospital admission.^1^ We searched conference abstracts from the International AIDS Society and Conferences on Retroviruses and Opportunistic Infections from 2016 onwards to identify studies that had been completed, but not yet published as full-text articles. Study inclusion was by consensus with disagreements resolved through discussion.

### Data analysis

Two reviewers (NF and AR), working independently in pairs, extracted data in accordance with a predefined data extraction sheet. If outcomes from the same cohort were published across different publications, each outcome was only reported once. Outcomes of interest were the proportion of patients who died, were readmitted to hospital, and were successfully linked to care after hospital discharge. We also extracted data about study setting, age, sex, length of hospital stay, diagnosis at index admission, history of previous admission, and CD4 cell count at discharge. Indicators of risk of bias were extracted using adapted items from the Newcastle-Ottawa scale.^16^ We calculated proportions and corresponding 95% CIs for all reported outcomes, and pooled data after transformation^17^, ^18^ using random-effects meta-analysis.^19^ We calculated a composite adverse outcome of death and hospital readmission. For studies in which loss to follow-up was reported, estimates were based on available cases. We did sensitivity analyses to assess differences in the proportion of patients who died following discharge or were readmitted to hospital by study location, duration of follow-up, advanced HIV disease status at admission, country economic status, and recency of publication (studies that reported outcomes up to the end of 2015 *vs* studies that reported outcomes from 2016 onwards); these subgroup proportions were compared using a two-sample z-test. Statistical tests for heterogeneity do not work well with pooled proportions, thus we assessed sources of heterogeneity through visual inspection of forest plot and exploration of outliers. We analysed all data with Stata (version 15.0).

### Role of the funding source

The funder of the study had no role in study design, data interpretation, or writing of the report.

## Results

**Figure 1:**
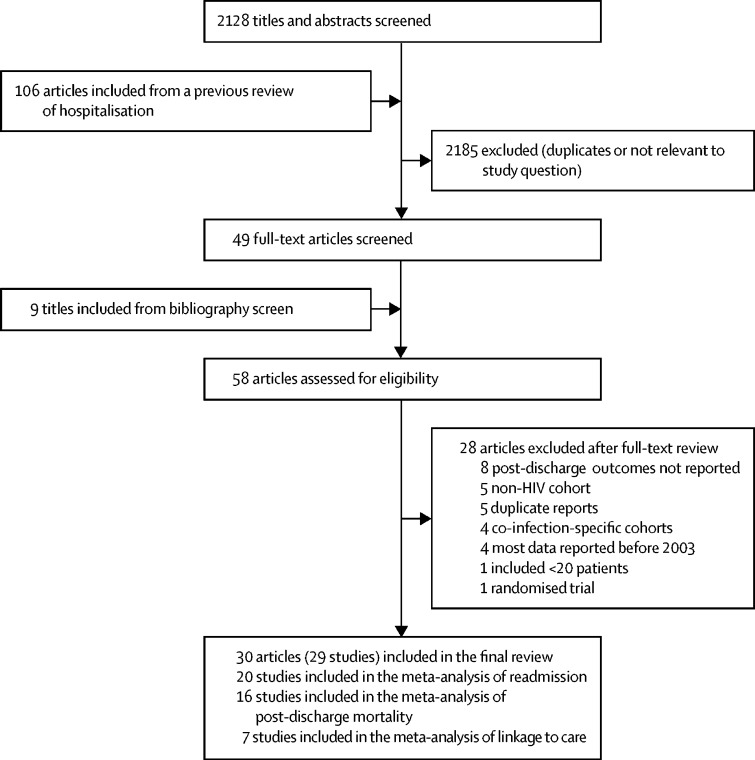
Study selection

From an initial screen of 2128 papers, 29 studies met the eligibility criteria and were included in the systematic review, 20 of which were included in the meta-analysis of readmission, six were included in the meta-analysis of post-discharge mortality, and seven were included in the meta-analysis of linkage to care (figure 1);^5^, ^11^, ^12^, ^13^, ^14^, ^20^, ^21^, ^22^, ^23^, ^24^, ^25^, ^26^, ^27^, ^28^, ^29^, ^30^, ^31^, ^32^, ^33^, ^34^, ^35^, ^36^, ^37^, ^38^, ^39^, ^40^, ^41^, ^42^, ^43^, ^44^ one study reported outcomes in two separate publications.^36^, ^37^ Two studies were excluded because they reported post-discharge outcomes only for patients who were admitted for cryptococcal meningitis.^45^, ^46^ One study was excluded because it reported outcomes among children admitted with complicated severe acute malnutrition.^47^ Another study was excluded as it was a randomised trial assessing early initiation of ART among severely ill patients admitted to an intensive care unit in Brazil that was terminated due to slow recruitment.^48^ The 29 studies provided information about post-discharge outcomes for 92 781 patients. Most studies were from North America;^11^, ^12^, ^13^, ^14^, ^20^, ^21^, ^24^, ^25^, ^29^, ^30^, ^31^, ^33^, ^34^, ^40^, ^44^ nine studies were from Africa, including five from South Africa,^5^, ^23^, ^27^, ^38^, ^43^ one from Malawi,^26^ one from Mozambique,^36^, ^37^ one from Tanzania,^41^ and one from Zambia;^32^ two studies were from southeast Asia;^22^, ^35^ one study was from Latin America;^28^ and two studies were from Europe (table).^39^, ^42^ The study from Mozambique reported outcomes in children;^36^, ^37^ all other studies were done in adults. 11 studies reported the proportion of patients with advanced HIV disease (CD4 count <200 cells per μL),^5^, ^11^, ^12^, ^13^, ^28^, ^29^, ^32^, ^35^, ^36^, ^37^, ^41^ which ranged from 16% to 91%. 11 studies reported length of hospital stay,^5^, ^11^, ^12^, ^13^, ^23^, ^26^, ^28^, ^29^, ^32^, ^43^, ^44^ which ranged from 4 days to 12 days.

**Table:** Studies included in the systematic review

	**Setting**	**Population**	**Study period**	**HIV-positive patients with outcomes, n**	**Men**	**Women**	**Age, years**	**Patients on ART, n (%)**	**CD4 count**		**Length of stay, days**	**Primary index admission**	**Post-discharge follow-up, days**	**Risk factors for readmission or post-discharge mortality**
									Median (IQR)	<200 cells per μL				
Alfandre et al^20^	USA	Adults	2012–13	33 270	17 896	15 374	>40 (81% of study population)	..	..	..	..	..	30	Discharged against medical advice
Antoniou et al^21^	Canada	Medically complex and socially vulnerable adults	2009–15	268	222	46	49	..	..	..	..	..	30	..
Ayudhya et al^22^	Thailand	Adults	2013	140*	76	44	40	..	160 (52–407)	..	..	..	30	..
Beckwith et al^23^	South Africa	Adults	2013–15	151	73	78	39	92 (61%)†	..	91% (<100 cells μL)	5	AIDS-related illness; tuberculosis	30	..
Berry et al^24^	USA	Adults	2013–15	45 382 admissions (33 556 individuals)	30 086	15 296	37% 45–54 (37% of study population)	..	..	..	..	Non-AIDS defining infection	30	..
Campbell et al^25^	USA	HIV-positive patients	2019	389	..		..	(78%)	..	..	..	..	30	..
Chawla et al^26^	Malawi	Adults	2013	1001	475	526	31–40 (38% of study population)	..	..	..	4	..	30	..
Cichowitz et al^27^	South Africa	Adults	2014	168 (136 HIV-positive at admission)	61	76	37	88 (65%)	56 (23–372)	..		..	180	..
Coelho et al^28^	Brazil	Adults	2007–13	1861 admitted to hospital; 1442 index hospitalisations	932	510	40	1080 (75%)	..	47%	12	AIDS-related illness	30	Low CD4 cell count at admission; longer length of stay; discharged against medical advice
Colasanti et al^29^	USA	Adults newly diagnosed with HIV in hospital	2011–12	94	72	22	43	0	134 (30–307)	..	10	..	730	..
Davy-Mendez et al^12^	USA	Adults (41% men who have sex with men)	1996–2016	2006	1376	630	37	..	..	16%	7	..	30	Low CD4 cell count at admission
English et al^30^	USA	Adults	2010–13	417	304	113	<50 (75% of study population)	..	..	65%	..	..		..
Gibson et al^14^	USA	Adults	2015–16	918	652	266	43		..	..	..	AIDS-related illness	30	Discharged against medical advice
Gupta and Dhanireddy^31^	USA	HIV-positive patients	2007–12	850	−	..	..	..	..	..	..	..	90	..
Haachambwa et al^32^	Zambia	Adults	2017–18	239	96	143	36	206 (86%)	181 (52–301)	..	12	Pulmonary (including tuberculosis)	74	
Hadlock et al^33^‡	USA	Adults	2010–14	908	..		48	..	..	55%	..	..	30	
Hoffmann et al^5^	South Africa	Adults	2016	121	54	67	40	81 (67%)	260 (113–464)	..	6	Pulmonary (including tuberculosis)	180	Longer length of stay; no linkage to care following discharge
Hsieh et al^34^	USA	Febrile adults who inject drugs	1998–2004	82	..	..	..	..	248 (2–1033)	..	..	..	90	
Khawcharoenporn et al^35^	Thailand	Adults	2013–15	240	138	102	37	103 (43%)	158 (72–382)	..	..	..-	30	
Madrid et al^36^, ^37^	Mozambique	Children	2000–16	258	..	..	1–5 (57% of study population)	..	..	..	..	..	90	
Meintjes et al^38^	South Africa	Adults	2012–13	585	247	338	35	263 (45%)	134 (53–275)	..		Tuberculosis (33·5%)	90	
Morquin et al^39^	France	Adults in intensive care unit	1997–2008	98	69	29	43	53 (54%)	..	55%	..	Respiratory and neurological failure	365	Low CD4 cell count at admission
Nijhawan et al^13^	USA	Adults	2011	930	..	..	45	..	..	62%	5·5	..	30	Not being on ART
Nijhawan et al^11^	USA	Adults	2006–08	1509	1102†	407†	43	..	..	52%	7	AIDS-related illness	30	
Parent et al^40^	Canada	Adults	1996–2015	7013	5649	1364	43	7013 (100%)	..	28%	..	..	30	Discharged against medical advice
Peck et al^41^	Tanzania	Adults	2013	143	47	58	47	105 (100%)	..	..	..	..	365	Longer length of stay or lack of linkage <1 month post discharge
Shaaban et al^42^	Portugal	Adults	2009–14	37 134	25 060	12 074	44	..	..	..	..	..	30	Discharged against medical advice
Stuart-Clark et al^43^	South Africa	Adults	2009	146	..		39	56 (38%)	111 (61–231)	..	6	..	365	
Tang et al^44^	USA	Adults	2009–12	185	..		50	..	..	..	5	..	30	..

ART=antiretroviral therapy.

* 140 patients admitted to hospital, but data on characteristics only provided for 120 patients**.**

† Estimated from overall sample (numbers not reported in original study).

‡ Characteristics provided for the 60 patients who were readmitted to hospital.

Overall, studies were rated as being at moderate risk of bias. 16 studies reported outcomes for all patients, 19 studies reported disaggregated outcomes per patient, and all 29 studies reported outcomes for at least 30 days after discharge. 13 studies reported vital status for all discharged patients and 12 studies either reported previous admission status or excluded patients with previous admissions from the study (appendix 3 p 2). There was moderate heterogeneity in outcomes, consistent with differences in disease severity at discharge, duration between discharge and readmission, and post-discharge planning and linkage to care. In subgroup analysis, outcomes also differed by geographical region and country income level.

Six studies (4487 admissions) reported in-hospital mortality; these studies were done in the USA,^12^, ^25^ South Africa,^5^, ^38^ Tanzania,^41^ and Zambia.^32^ Mortality prior to discharge ranged from 1·7% (95% CI 1·2–2·3) to 26·7% (19·9–34·2), indicating substantial heterogeneity, with an overall pooled proportion of 6·1% (3·1–9·1).

Seven studies^5^, ^21^, ^22^, ^29^, ^30^, ^35^, ^41^ (1296 admissions) reported the proportion of patients who were successfully linked to care after hospital discharge (defined as linkage to HIV care, outpatient care, or community care). The proportion of patients who were successfully linked to care after discharge ranged from 10·9% (95% CI 7·0–15·5) to 79·1% (71·5-85·8%), with an overall pooled proportion of 41·6% (23·6–59·6).

**Figure 2:**
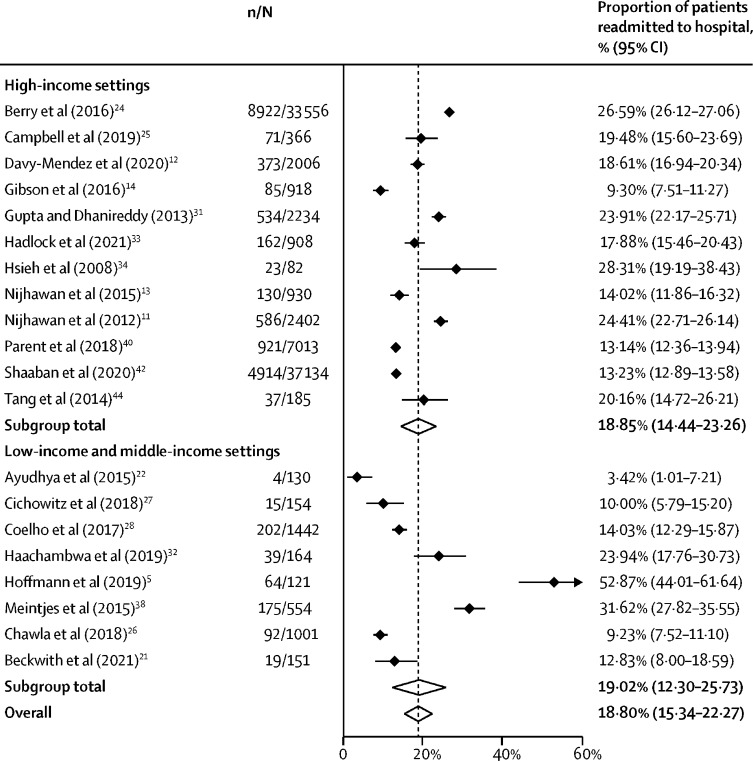
Post-discharge readmission by study setting n=number of readmissions. N=total number of patients. Black diamonds show point estimates, white diamonds show pooled estimates, and the dashed line shows the overall pooled estimate.

**Figure 3:**
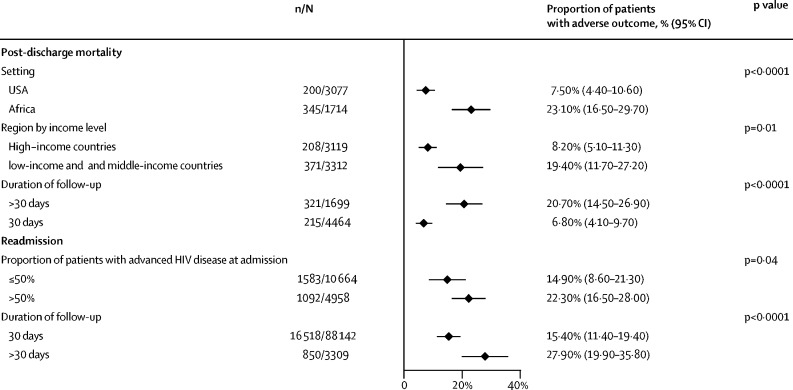
Post-discharge mortality and readmissions by setting, region, duration of follow-up, and advanced HIV status at admission n=number of events. N=total number of patients.

20 studies (91451 admissions) reported the number of patients readmitted to care after hospital discharge,^5^, ^11^, ^12^, ^13^, ^14^, ^22^, ^23^, ^24^, ^25^, ^26^, ^27^, ^28^, ^31^, ^32^, ^33^, ^34^, ^38^, ^40^, ^42^, ^44^ of which, 14 studies reported readmission within 30 days of discharge. The proportion of patients readmitted post-discharge ranged from 3·4% (1·0–7·2) to 52·9% (44·0–61·6), with an overall pooled proportion of 18·8% (15·3–22·3; figure 2). The pooled proportion of patients readmitted to hospital was also higher for studies that reported readmissions that occurred after 30 days (figure 3). One study reported that the risk of readmission within 30 days of discharge was higher for people living with HIV who had been discharged against medical advice; however, there were insufficient data to include this in the meta-analysis.^20^

In sensitivity analysis, no differences were identified in the proportion of patients readmitted to hospital comparing studies published before 2016 and those published after 2016, when universal ART was introduced. No differences in the proportion of patients readmitted to hospital were identified when comparing studies from the USA and Africa, and high-income countries compared with low-income and middle-income settings. The proportion of readmissions was higher in studies in which the majority of patients had advanced HIV disease at admission than those in which less than half of patients had advanced HIV disease at admission (figure 3).

**Figure 4:**
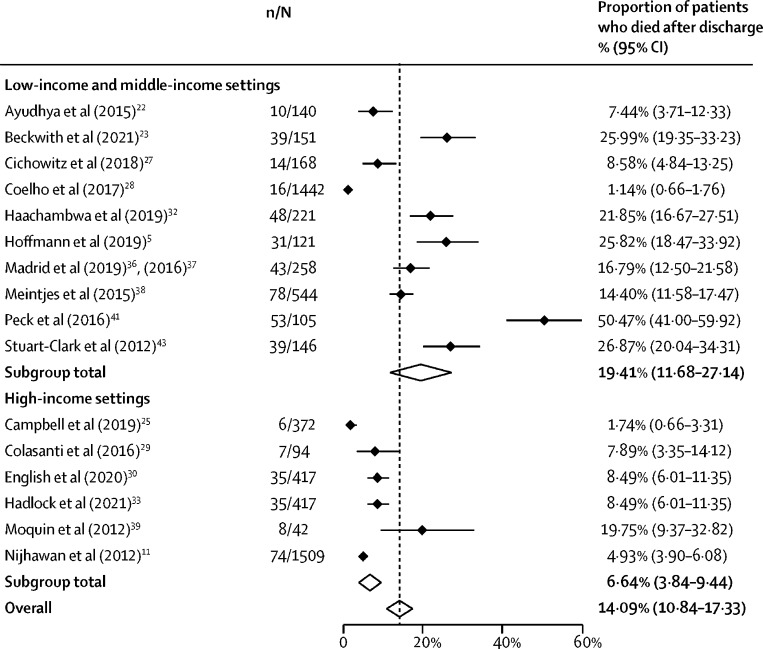
Post-discharge mortality by study setting n=number of deaths. N=total number of patients. Black diamonds show point estimates, white diamonds show pooled estimates, and the dashed line shows the overall pooled estimate.

16 studies (6163 patients) reported the number of patients who died after discharge.^5^, ^13^, ^22^, ^23^, ^25^, ^27^, ^28^, ^29^, ^30^, ^32^, ^33^, ^36^, ^38^, ^39^, ^41^, ^43^ The proportion of patients who died after discharge ranged from 1·1% (95% CI 0·7–1·7) to 50·5% (41·0–59·9), with an overall pooled proportion of 14·1% (10·8–17·3) (figure 4). In sensitivity analysis, no difference was identified in the proportion of patients who died following discharge when comparing studies published before 2016 and studies published after 2016. Post-discharge mortality was higher in studies from Africa than studies from the USA, and from studies done in low-income countries and middle-income countries compared with high-income countries. Post-discharge mortality was higher in studies in which people were followed up for longer than 30 days (figure 3).

11 studies (6598 admissions) provided data on both the number of patients who were readmitted and the number who died following discharge.^5^, ^11^, ^21^, ^22^, ^23^, ^25^, ^27^, ^28^, ^32^, ^33^, ^38^ The proportion of patients with this composite adverse outcome ranged from 11·0% (95% CI 6·3–17·0) to 78·3% (70·6–85·1), with a pooled proportion of 31·7% (23·9–39·5).

Post-discharge mortality was associated with a lower CD4 cell count^11^, ^39^ and lack of linkage to care within 1 month after discharge.^41^ A US study reported that inpatient treatment with ART during hospitalisation reduced the risk of short-term mortality.^25^ A study from Tanzania reported that more than half of adults living with HIV had died within 12 months of hospital discharge, and nearly a third had died within the first 3 months, with a 2 times higher adjusted risk of post-discharge mortality compared with HIV-negative adults.^41^ The high mortality in this study might be the result of the condition of advanced HIV disease among individuals who were admitted, and although this study did not report information on CD4 cell count, early linkage to primary HIV care was associated with a 75% lower incidence of post-discharge mortality. Reported risk factors for readmission included low CD4 cell count at admission,^12^, ^28^ longer length of stay,^5^, ^28^ not being on ART,^13^ discharge against medical advice,^14^, ^20^, ^28^, ^40^, ^42^ and not linking to care after discharge.^5^ A US study of febrile people who inject drugs reported a higher rehospitalisation rate among those with newly diagnosed HIV infection than those with known HIV infection (40% *vs* 26%), highlighting the importance of tailored support for this patient group as part of discharge plans.^34^

Six studies reported outcomes for people living with HIV and those who were HIV-negative. In Tanzania, 28·9% of adults with HIV-infected died within the first 3 months after discharge compared with 17·7% of HIV-negative adults.^41^ In Mozambique, children with HIV had a higher rate of post-discharge mortality than did children without HIV (hazard ratio [HR] 1·77 [95% CI 1·07–2·91]).^36^, ^37^ Two US studies found that HIV infection was associated with readmission. The first study found the risk of readmission was 1·5 times higher (95% CI 1·46–1·54) among adults with HIV than those without HIV.^24^ The second study was among people who inject drugs and reported a HR of 2**·**90 (1**·**20–7**·**02) for readmission comparing people with HIV with those without HIV.^34^ A study from Malawi reported that patients with HIV who were discharged from hospital had a higher risk of readmission than HIV-negative individuals (adjusted risk ratio 2·41 [95% CI 1·64–3·53]).^26^ In contrast, a study from South Africa found no difference in readmission, death, or loss to follow-up by HIV status.^27^

## Discussion

This systematic review summarises outcomes of a population of people living with HIV at high risk of death. We found that almost a third of patients with HIV had an adverse outcome after hospital discharge, (mortality or readmission to hospital), mostly within 30 days of discharge. Where comparative information by serostatus was available, outcomes were generally worse for people living with HIV. This finding is consistent with a previous study, not included in this review, among children admitted with complicated severe acute malnutrition in Zambia and Zimbabwe; in this study, children who were HIV-positive had an almost four times higher risk of post-discharge mortality than HIV-negative children, regardless of ART initiation.^47^ This finding represents a missed opportunity to reduce HIV-associated mortality, and results in increased utilisation of health-care resources. Post-discharge mortality was higher in low-income and middle-income countries, in particular when comparing Africa and the USA. No apparent decrease was observed in the proportion of people with HIV who had adverse outcomes post-discharge when comparing studies done before 2016 with those done from 2016 onwards, when initiation of ART is recommended as soon as a positive HIV diagnosis is confirmed.^49^

The majority of adverse outcomes occurred within 30 days following discharge, but the proportion of patients who had an adverse outcome overall was higher in studies that followed up patients for longer durations. 30-day readmission rate is commonly used as an indicator for quality of hospital care, but follow-up over a longer time period provides a better estimate of adverse events after discharge and helps to identify a larger proportion of patients at risk of mortality and readmission. Programmes should consider patient follow-up after the 30-day discharge period to account for adverse outcomes and to enable intervention to reduce such outcomes, similar to that recommended for other areas of patient care.^50^

Several studies included provide insights into risk factors for adverse outcomes and potential interventions to reduce mortality and readmission following discharge. In subgroup analyses, readmissions were more common in studies in which a higher proportion of index admissions had advanced HIV disease. Several studies reported that mortality post-discharge was associated with a lower CD4 cell count,^11^, ^38^ whereas initiation of ART in hospital^25^ was associated with decreased mortality. Risk factors for readmission included discharge against medical advice,^14^, ^20^, ^28^ not being on ART,^13^ having an AIDS-defining illness,^11^ low CD4 cell count,^12^, ^28^ high viral load,^12^ and no support for linkage to care.^5^ One study reported that 18% of patients on ART were readmitted within 30 days compared with 26% of patients who were not on ART, highlighting the importance of ensuring patients who are not on ART and have no contraindications are started on treatment before being discharged.^25^

The provision of patient support for linkage to care after discharge is an important strategy to reduce readmission and improve outcomes. This systematic review included a study from Thailand, which found that an intervention bundle including enhanced care during admission and appointment scheduling and reminders post-discharge was associated with significantly higher HIV care engagement post-discharge.^35^ These observations are consistent with findings from a systematic review, which found that patient-centred discharge instructions and telephone follow-up calls were common components of effective intervention bundles to reduce 30-day readmission.^51^

We used a broad search to identify a large number of studies reporting readmission and death after discharge across a range of settings. Post-discharge mortality was higher in studies that reported outcomes over a longer follow-up period, suggesting that the pooled estimate of post-discharge mortality would be higher if studies had longer follow-up periods. There are several other limitations to note. Because this systematic review was not primarily focused on mortality during admission, the reported estimates are not representative of all studies reporting this outcome—eg, a previous systematic review of causes of hospital admission found that mortality among individuals admitted to hospital was 20% for adults and 14% for children.^1^ Importantly, many programmes will underestimate negative post-discharge outcomes because these outcomes have not been detected in routine information systems or patients attended a clinic outside the catchment area of the information system. Reporting from research settings that have interventions in place to improve ascertainment of outcomes might not be representative of outcomes within routine programmes with fewer resources. We investigated risk of readmission post-discharge, but it is likely that some of the index admissions were in fact readmissions from a recent hospital discharge. Previous hospitalisation, a risk factor for readmission,^52^ was infrequently reported but might explain some of the adverse outcomes reported. Palliative care provision was not reported; anticipated deaths following discharge to palliative care might have occurred but were not reported and their inclusion effectively misclassifies post-discharge mortality as an adverse outcome.^53^ Most studies included were from high-income countries and reported outcomes among adults; no studies from west and central Africa, and few studies from southeast Asia or the Western Pacific region were included. Although this paucity of reported information from these regions does not necessarily represent publication bias, it does highlight the need for data from a broader set of patient populations and countries. A number of studies did not account for outcomes among patients who were lost to follow-up after discharge, thus estimates of adverse outcomes are likely to be an underestimate, particularly because studies were unable to correct status ascertainment through linkage to vital registration systems.^54^, ^55^ Outcomes were inconsistently reported by the studies, with only around a quarter of studies reporting linkage to care and around a half reporting mortality after discharge. Key study characteristics were also inconsistently reported, such as length of hospital stay, CD4 cell count, and ART status.

In conclusion, this systematic review found high rates of mortality and readmission after hospital discharge of people living with HIV, highlighting the importance of ensuring post-discharge referral and appropriate post-discharge management to improve outcomes among these vulnerable patients. Further research and investment is needed to identify feasible approaches targeting individuals at highest risk of poor outcomes after discharge and to provide adapted support to ensure appropriate linkage and continuity of support post-discharge, including ART.

## Date sharing

All data are available in the original published articles included in this systematic review, and are available upon request from the corresponding author.

## Declaration of interests

We declare no competing interests.

## Supplementary Material

French translation of the abstract

Spanish translation of the abstract
