# Supplementary material for: Outcomes of people living with HIV after hospital discharge: a systematic review and meta-analysis
Source: Lancet HIV. 2022 Mar 1;9(3):e150–9. doi: 10.1016/S2352-3018(21)00329-5 (PMC8905089; doi:10.1016/S2352-3018(21)00329-5)
Supplement: French translation of the abstract [file mmc1.pdf]

THE LANCET
HIV

Supplementary appendix 1

This translation in French was submitted by the authors and we reproduce it as supplied. It has not been peer reviewed. *The Lancet*'s editorial processes have only been applied to the original in English, which should serve as reference for this manuscript.

Cette traduction en français a été proposée par les auteurs et nous l'avons reproduite telle quelle. Elle n'a pas été examinée par des pairs. Les processus éditoriaux du *Lancet* n'ont été appliqués qu'à l'original en anglais et c'est cette version qui doit servir de référence pour ce manuscrit.

Résultats des personnes vivant avec le VIH après leur sortie de l'hôpital: une revue systématique et une méta-analyse

Contexte L'identification et la prise en charge adéquate des personnes vivant avec le VIH à un stade avancé de la maladie est un élément clé de la lutte contre le VIH. Les personnes vivant avec le VIH qui sont hospitalisées courent un niveau de risque accru de décès, un risque qui peut persister après la sortie de l'hôpital. Les objectifs de cette étude étaient d'estimer la fréquence des résultats négatifs après la sortie de l'hôpital et de déterminer les facteurs de risque de ces résultats chez les personnes vivant avec le VIH.

Méthodes En utilisant une stratégie de recherche large combinant les termes pour la sortie de l'hôpital et l'infection par le VIH, nous avons effectué une recherche dans MEDLINE à travers PubMed et Embase, du 1er janvier 2003 au 30 novembre 2021 pour identifier les études fournissant des résultats chez les personnes vivant avec le VIH après la sortie de l'hôpital. Nous avons estimé les proportions regroupées de réadmissions et de décès après la sortie de l'hôpital à l'aide de modèles à effets aléatoires. Nous avons également effectué des analyses de sous-groupes par contexte, région, durée du suivi et sérologie du VIH avancé à l'admission, ainsi que des analyses de sensibilité pour évaluer l'hétérogénéité.

Résultats Nous avons obtenu 29 cohortes qui ont rapporté les résultats des personnes vivant avec le VIH après la sortie de l'hôpital parmi 92 781 patients. La proportion regroupée de patients réadmis après la sortie était de 18,8 % (15,3-22,3 %) et 14,1 % (10,8-17,3 %) sont décédés après la sortie. Dans les analyses de sensibilité, aucune différence n'a été identifiée dans la proportion de patients réadmis ou décédés lors de la comparaison des études publiées avant 2016 avec celles publiées après 2016. La mortalité après la sortie de l'hôpital était plus élevée dans les études effectuées en Afrique (23,1 %, 16,5-29,7 %) que dans celles effectuées aux États-Unis (7,5 %, 4,4-10,6 %). Pour les études qui ont rapporté à la fois la mortalité après la sortie et la réadmission, la proportion regroupée de patients qui ont eu ce critère défavorable composite était de 31,7 % (23,9-39,5 %). L'hétérogénéité était modérée et s'expliquait en grande partie par l'état du patient et son lien avec les soins. Les facteurs de risque signalés pour la réadmission comprenaient un faible nombre de cellules CD4 à l'admission, une durée de séjour plus longue, une sortie contre l'avis médical et l'absence de lien avec les soins après la sortie; le traitement avec les antirétroviraux pendant l'hospitalisation était un facteur protecteur de la mortalité après la sortie de l'hôpital.

Interprétation Plus d'un quart des patients atteints de VIH ont eu un résultat défavorable après la sortie de l'hôpital sans aucun signe d'amélioration au cours des 15 dernières années. Cette revue souligne l'importance de garantir une recommandation orientation a la sortie de l'hôpital et une prise en charge appropriée, y compris l'administration d'antirétroviraux, afin de réduire la mortalité et la réhospitalisation parmi ce groupe de patients à haut risque.
