# Supplementary material for: Outcomes of people living with HIV after hospital discharge: a systematic review and meta-analysis
Source: Lancet HIV. 2022 Mar 1;9(3):e150–9. doi: 10.1016/S2352-3018(21)00329-5 (PMC8905089; doi:10.1016/S2352-3018(21)00329-5)
Supplement: Spanish translation of the abstract [file mmc2.pdf]

THE LANCET
HIV

Supplementary appendix 2

This translation in Spanish was submitted by the authors and we reproduce it as supplied. It has not been peer reviewed. *The Lancet*'s editorial processes have only been applied to the original in English, which should serve as reference for this manuscript.

Los autores nos proporcionaron esta traducción al español y la reproducimos tal como nos fue entregada. No la hemos revisado. Los procesos editoriales de *The Lancet* se han aplicado únicamente al original en inglés, que debe servir de referencia para este manuscrito.

Resultados tras el alta hospitalaria de las personas que viven con VIH : una revisión sistemática y un metanálisis

Antecedentes La identificación y el manejo adecuado de las personas en la fase más avanzada de la infección por el VIH es un componente clave de la respuesta al VIH. Las personas con VIH que están hospitalizadas presentan un mayor riesgo de muerte, que puede persistir después del alta. Este estudio tenía como objetivos de este estudio estimar la frecuencia de resultados negativos después del alta y determinar los factores de riesgo en personas con VIH.
Métodos Utilizamos una estrategia de búsqueda amplia que combinaba términos para alta hospitalaria e infección por VIH. Buscamos en MEDLINE a través de PubMed y Embase desde el 1 de enero de 2003 hasta el 30 de noviembre de 2021 para identificar estudios que informan sobre resultados en personas con VIH tras el alta hospitalaria. Se estimaron las proporciones agrupadas de reingresos y muerte posterior al alta hospitalaria mediante modelos de efectos aleatorios. También realizamos análisis de subgrupos por ámbito, región, duración del seguimiento y presencia de VIH avanzado al ingreso. Realizamos análisis de sensibilidad para evaluar la heterogeneidad.
Hallazgos Obtuvimos 29 cohortes que presentaban resultados tras el alta hospitalaria en 92 781 personas que viven con el VIH. La proporción agrupada de pacientes readmitidos después del alta fue del 18,8% (15,3-22,3%) y el 14,1% (10,8-17,3%) falleció después del alta. En los análisis de sensibilidad no se identificaron diferencias en la proporción de pacientes que reingresaron o fallecieron al comparar estudios publicados antes de 2016 con los publicados después de 2016. La mortalidad posterior al alta fue mayor en los estudios de África (23,1%, 16,5-29,7%) en comparación con EE.UU. (7,5%, 4,4-10,6%). Para los estudios que informaron tanto sobre el reingreso como sobre la mortalidad posterior al alta, la proporción de pacientes que tuvieron este resultado adverso combinado fue del 31,7 % (23,9-39,5 %). La heterogeneidad fue moderada y se explica en gran medida por el estado de los pacientes y su vinculación con la atención. Los factores de riesgo para la readmisión de los que se informó incluían un recuento bajo de células CD4 al momento de la admisión, una estadía más prolongada, el alta en contra del consejo médico y la falta de vinculación con la atención después del alta. Recibir tratamiento antirretroviral (TARV) durante la hospitalización fue un factor protector de la mortalidad posterior al alta.
Interpretación Más de una cuarta parte de los pacientes con VIH tuvieron un resultado adverso después del alta hospitalaria, sin evidencia de mejoría en los últimos 15 años. Esta revisión destaca la importancia de garantizar la derivación posterior al alta y el manejo adecuado, incluido el TARV, para reducir la mortalidad y la rehospitalización en este grupo de pacientes de alto riesgo.
