# Supplementary material for: Outcomes of people living with HIV after hospital discharge: a systematic review and meta-analysis
Source: Lancet HIV. 2022 Mar 1;9(3):e150–9. doi: 10.1016/S2352-3018(21)00329-5 (PMC8905089; doi:10.1016/S2352-3018(21)00329-5)
Supplement: Supplementary appendix [file mmc3.pdf]

THE LANCET
HIV

Supplementary appendix 3

This appendix formed part of the original submission and has been peer reviewed. We post it as supplied by the authors.

Supplement to: Ford N, Patten G, Rangaraj A, et al. Outcomes of people living with HIV after hospital discharge: a systematic review and meta-analysis. *Lancet HIV* 2022; **9:** 150–59.

Appendix

1. **Study quality for the outcome of 30-day readmission**
2. **Systematic Review Protocol**

1. Table. Study quality for outcome of 30-day readmission

Study	Prospective design	Outcomes reported for all patients	At least 30 days follow up post discharge	Prior admission accounted for	Post discharge mortality fully accounted for	Outcomes disaggregated per patient (not per admission)
Alfrandre[1]	N	Y	Y	N	N	N
Antoniou[2]	Y	N	Y	Y	N	Y
Ayudhya[3]	Y	N	Y	Y	N	Y
Beckworth[4]	N	N	Y	N	Y	Y
Berry[5]	N	Y	Y	Y	N	N
Campbell[6]	Y	Y	Y	N	Y	N
Chawla[7]	N	Y	Y	Y	Y	Y
Cichowitz[8]	Y	N	Y	Y	Y	Y
Coelho[9]	N	Y	Y	N	Y	N
Colasanti[10]	N	Y	Y	N	N	Y
Davy-Mendez[11]	N	N	Y	N*	N	N
English[12]	Y	Y	Y	N	Y	Y
Gibson[13]	N	Y	Y	N	N	Y
Gupta[14]	N	Y	Y	N	N	N
Haachambwa[15]	Y	N*	Y	N	Y	Y
Hadlock[16]	N	N	Y	Y	N	Y
Hoffmann[17]	Y	Y	Y	N	Y	Y
Hsieh[18]	N	N	Y	N	N	Y
Khawcharoenporn[19]	Y	N	Y	N	Y	Y
Madrid[20,21]	N	N	Y	N	Y	N
Meintjes[22]	Y	Y	Y	Y	Y	Y
Morquin[23]	N	N	Y	Y	N	Y
Nijhawan[24]	N	Y	Y	Y	N	N
Nijhawan[25]	N	N	Y	Y	Y	N
Parent[26]	N	Y	Y	Y	N	Y
Peck[27]	Y	Y	Y	Y	Y	Y
Shaaban[28]	N	Y	Y	N	N	N
Stuart-Clark[29]	Y	N	Y	N	N	Y
Tang[30]	N	Y	Y	N	N	Y

[#] Study discontinued early due to poor recruitment
* Index hospitalization defined as no hospitalization in the prior 30 days

References

1. Alfandre D, Yang J, Harwood K, et al. "Against Medical Advice" Discharges Among HIV-Infected Patients: Health and Health Services Outcomes. *The Journal of the Association of Nurses in AIDS Care : JANAC* 2017; **28**(1): 95-104.
2. Antoniou T, Graves E, Plumptre L, Stewart A, Carusone SC. Antiretroviral prescription pick-up and physician follow-up after hospital discharge among medically complex people with HIV. *Open Forum Infectious Diseases* 2019; **6**(2).
3. Ayudhya DPN, Khawcharoenporn T. HIV care engagement within 30 days after hospital discharge among patients from a Thai tertiary-care centre. *International Journal of STD and AIDS* 2015; **26**(7): 467-9.
4. Beckwith P, Tlali M, Charalambous S, et al. Causes and Outcomes of Admission and Investigation of Tuberculosis in Adults with Advanced HIV in South African Hospitals: Data from the TB Fast Track Trial. *Am J Trop Med Hyg* 2021.
5. Berry SA, Fleishman JA, Moore RD, Gebo KA. Thirty-day hospital readmissions for adults with and without HIV infection. *HIV Medicine* 2016; **17**(3): 167-77.
6. Campbell J, Polk C, Roshdy D, Leonard M. Inpatient initiation of art improves short-term mortality in people living with HIV. *Open Forum Infectious Diseases* 2019; **6**: S479.

7. Chawla KS, Rosenberg NE, Stanley C, et al. HIV and early hospital readmission: evaluation of a tertiary medical facility in Lilongwe, Malawi. *BMC Health Serv Res* 2018; **18**(1): 225.
8. Cichowitz C, Pellegrino R, Motlhaoleng K, Martinson NA, Variava E, Hoffmann CJ. Hospitalization and post-discharge care in South Africa: A critical event in the continuum of care. *PLoS One* 2018; **13**(12): e0208429.
9. Coelho LE, Ribeiro SR, Japiassu AM, et al. Thirty-day Readmission Rates in an HIV-infected Cohort from Rio de Janeiro, Brazil. *Journal of Acquired Immune Deficiency Syndromes* 2017; **75**(4): e90-e8.
10. Colasanti J, Goswami ND, Khoubian JJ, et al. The perilous road from HIV diagnosis in the hospital to viral suppression in the outpatient clinic. *AIDS Research and Human Retroviruses* 2016; **32**(8): 729-36.
11. Davy-Mendez T, Napravnik S, Wohl DA, et al. Hospitalization rates and outcomes among persons living with human immunodeficiency virus in the southeastern united states, 1996-2016. *Clinical Infectious Diseases* 2020; **71**(7): 1616-23.
12. English K, May SB, Davila JA, et al. Retention in Care and Viral Load Improvement After Discharge Among Hospitalized Out-of-Care People With HIV Infection: A Post Hoc Analysis of a Randomized Controlled Trial. *Open Forum Infect Dis* 2020; **7**(6): ofaa193.
13. Gibson MP, Nijhawan AE, Jain MK, Halm E. Thirty-day readmissions among HIV-infected individuals at a safety-net hospital: Predictors and preventability. *Open Forum Infectious Diseases* 2016; **3**.
14. Gupta R, Dhanireddy S. Hospital readmissions among indigent HIV-infected patients: A movement toward medical home model interventions. *Journal of General Internal Medicine* 2013; **28**: S90-S1.
15. Haachambwa L, Kandiwo N, Zulu PM, et al. Care Continuum and Postdischarge Outcomes among HIV-Infected Adults Admitted to the Hospital in Zambia. *Open Forum Infectious Diseases* 2019; **6**(10).
16. Hadlock GC, Moleres KA, Pineda LJ, Jakeman B. Risk factors for potentially preventable hospital readmissions among persons living with human immunodeficiency virus infection. *AIDS Care* 2021; **33**(3): 306-10.
17. Hoffmann CJ, Milovanovic M, Cichowitz C, Kinghorn A, Martinson NA, Variava E. Readmission and death following hospitalization among people with HIV in South Africa. *PLoS ONE* 2019; **14**(7).
18. Hsieh YH, Rothman RE, Bartlett JG, Yang S, Kelen GD. HIV seropositivity predicts longer duration of stay and rehospitalization among nonbacteremic febrile injection drug users with skin and soft tissue infections. *J Acquir Immune Defic Syndr* 2008; **49**(4): 398-405.
19. Khawcharoenporn T, Damronglerd P, Chunloy K, Sha BE. Enhanced inpatient rounds, appointment reminders, and patient education improved HIV care engagement following hospital discharge. *Int J STD AIDS* 2018; **29**(7): 641-9.
20. Madrid L, Casellas A, Sacoor C, et al. Postdischarge mortality prediction in sub-Saharan Africa. *Pediatrics* 2019; **143**(1).
21. Madrid L, Sitoe A, Aldea M, et al. Mortality following discharge in children admitted to a rural mozambican hospital: Development of a prediction model to identify children at risk of death. *American Journal of Tropical Medicine and Hygiene* 2016; **95**(5): 531.
22. Meintjes G, Kerkhoff AD, Burton R, et al. HIV-Related Medical Admissions to a South African District Hospital Remain Frequent Despite Effective Antiretroviral Therapy Scale-Up. *Medicine (Baltimore)* 2015; **94**(50): e2269.
23. Morquin D, Le Moing V, Mura T, et al. Short- and long-term outcomes of HIV-infected patients admitted to the intensive care unit: impact of antiretroviral therapy and immunovirological status. *Ann Intensive Care* 2012; **2**(1): 25.
24. Nijhawan AE, Kitchell E, Etherton SS, Duarte P, Halm EA, Jain MK. Half of 30-Day Hospital Readmissions among HIV-Infected Patients Are Potentially Preventable. *AIDS Patient Care and STDs* 2015; **29**(9): 465-73.
25. Nijhawan AE, Clark C, Kaplan R, Moore B, Halm EA, Amarasingham R. An electronic medical record-based model to predict 30-day risk of readmission and death among HIV-infected inpatients. *J Acquir Immune Defic Syndr* 2012; **61**(3): 349-58.
26. Parent S, Barrios R, Nosyk B, et al. Impact of Patient-Provider Attachment on Hospital Readmissions Among People Living With HIV: A Population-Based Study. *J Acquir Immune Defic Syndr* 2018; **79**(5): 551-8.

27. Peck RN, Wang RJ, Mtui G, et al. Linkage to primary care and survival after hospital discharge for HIV-infected adults in Tanzania: a prospective cohort study. *Journal of Acquired Immune Deficiency Syndromes* 2016.
28. Shaaban AN, Dias SS, Muggli Z, Peleteiro B, Martins MRO. Risk of Readmission Among HIV Patients in Public Portuguese Hospitals: Longitudinal Multilevel Population-Based Study. *Front Public Health* 2020; **8**: 15.
29. Stuart-Clark H, Vorajee N, Zuma S, et al. Twelve-month outcomes of patients admitted to the acute general medical service at Groote Schuur Hospital. *S Afr Med J* 2012; **102**(6): 549-53.
30. Tang N, Maselli JH, Gonzales R. Variations in 30-day hospital readmission rates across primary care clinics within a tertiary referral center. *J Hosp Med* 2014; **9**(11): 688-94.

2. Systematic Review Protocol: Outcomes of PLHIV following hospital discharge: a systematic review

Contact
Nathan Ford, WHO, Geneva
Email: fordn@who.int

1. INTRODUCTION

The identification and management of people with advanced HIV disease is a key component in the HIV response. Hospitalizations from complications relating to HIV infection, including co-infections associated with advanced HIV, remain substantial. A recent review found that AIDS-related infections and bacterial infections are leading causes of hospital admission. Low CD4 cell count and low antiretroviral coverage at admission are major contributors to this disease profile and associated mortality.[1,2]

People who are hospitalized are at a heightened risk of death.[2-6] This risk that may persist after hospital discharge,[7] and people living with HIV who survive to hospital discharge have been described as a population with high mortality.[5]

A study from South Africa found that even with widespread access to antiretroviral therapy, the majority of inpatient natural deaths were likely related to HIV.[8] HIV is also commonly associated with readmission following discharge.[9] Several studies have identified factors associated with poor post-discharge outcomes among people living with HIV including readmission, failed linkage to care and death. These include low CD4 cell count[10], lack of antiretroviral therapy,[11] and discharge against medical advice.[12]

We did this systematic review and meta-analysis to assess post-discharge outcomes of people living with HIV who are discharged and summarize risk factors associated with poor outcomes.

The objective of this systematic review is to assess the available evidence on post-discharge outcomes of PLHIV.

2. METHODS

2.1. Types of studies

- Randomized and quasi-randomized controlled trials
- Comparative and non-comparative observational studies
- Other studies designs can be included if they contain relevant quantitative data

2.2. Types of participants

Inclusions

- HIV positive adults and children discharged from hospital

Exclusions

- Studies in which <20 patients were included

- Studies where outcomes could not be disaggregated according to HIV status
- Studies of patients admitted for a specific comorbidity that is unrelated to HIV
- Studies in which the majority (>50%) of outcome data is reported prior to 2003 – the period in which ART began to be available at scale in resource-limited settings

2.3. Types of outcomes

Primary outcomes post discharge:

- Number readmitted
- Number died
- Number successfully linked to care

Post-discharge mortality and re-admission to care will also be reported as a composite adverse outcome.

Secondary outcomes:

- Number of patients who died during hospitalization

2.4. Databases

The following databases will be searched.

- Embase
- Medline via Pubmed

In addition, all Conferences of the International AIDS Society (IAS) and Conferences on Retroviruses and Opportunistic Infections (CROI) will be searched from 2016 onwards to identify studies that have been completed but not yet published as full text.

2.6. Restrictions

No date, language, or geographical exclusions will be applied.

3.0. Risk of bias

Risk of bias will be assessed using and adapted Newcastle Ottawa score for all studies (for the purposes of this review, any information included from randomized trials will be treated as coming from observational studies.

4.0. Quantitative synthesis

Outcome data will be pooled using the DerSimonian-Laird random-effects method following appropriate transformation of the raw proportions.[13] Heterogeneity will be assessed through visual inspection of forest plots and subgroup analysis exploring outcomes differences by proportion with advanced HIV disease at admission, setting, World Bank income level, and duration of follow up.

Search strategies

PUBMED

#		Results 11/30/2021
#1	(Hospitalization [mesh] OR hospitaliz*[tiab] OR hospitalis*[tiab] OR re-hospital*[tiab] OR rehospital*[tiab] OR readmiss*[tiab] OR re-admiss* OR readmit*[tiab] OR re-admit*[tiab] OR admit*[tiab] OR admis*[tiab])	789,229
#2	"Patient Discharge"[Mesh] OR patient-discharge*[tiab] OR Patients-discharg*[tiab] OR post-discharg*[tiab] OR postdischarg*[tiab] OR discharge-plan*[tiab] OR hospital-discharg*[tiab] OR posthospital*[tiab] OR post-hospital*[tiab] OR after-discharg*[tiab] OR following-discharg* OR recent-dischar* OR following-hospitali*[tiab] OR recent-hospital*[tiab] OR after-hospital*[tiab] OR discharge-follow-up*[tiab] OR discharge-followup*[tiab] OR hospital-to-home-discharg*[tiab] OR (Discharg*[tiab] AND hospital*[tiab])	165,031
#3	"HIV Infections" [MeSH] OR "HIV"[MeSH] OR "hiv"[tw] OR "hiv1"[tw] OR "hiv2"[tw] OR "human immunodeficiency virus"[tw] OR "human immunedeficiency virus"[tw] OR "human immuno-deficiency virus"[tw] OR "human immune-deficiency virus"[tw] OR ((human -immun*) AND ("deficiency virus"[tw])) OR "acquired immunodeficiency syndrome"[tw] OR "acquired immunedeficiency syndrome"[tw] OR "acquired immuno-deficiency syndrome"[tw] OR "acquired immune-deficiency syndrome"[tw] OR ((acquired immun*) AND ("deficiency syndrome"[tw])) OR "Sexually Transmitted Diseases, Viral"[MeSH:NoExp] OR "HIV Long-Term Survivors"[Mesh] OR AIDS-virus*[tw]	434,444
#5	#1 AND #2 AND #3	1,219

2. Embase

#		Results 11/30/2021
#1	'hospital readmission'/exp OR readmiss* OR readmitt* OR rehospital* OR re?admiss* OR re?admitt* OR re?hospital* OR 'hospital admission'/exp OR 'child hospitalization'/exp OR ((hospital* OR patient*) NEAR/3 (admiss* OR admitt*))	580,891
#2	'hospital discharge'/exp OR discharg* NEAR/3 hospital*	191,614
#3	'human immunodeficiency virus infection'/exp OR 'human immunodeficiency virus'/exp OR hiv:ti OR hiv:ab OR 'hiv-1':ti OR 'hiv-1':ab OR 'hiv-2':ti OR 'hiv-2':ab OR 'human immunodeficiency virus':ti OR 'human immuno deficiency':ab OR 'human immuno-deficiency virus':ti OR 'human immuno-deficiency virus':ab OR 'human immunedeficiency virus':ti OR 'human immune deficiency virus':ab OR 'human immune-deficiency virus':ti OR 'human immune-deficiency virus':ab OR 'acquired immune-deficiency syndrome':ti OR 'acquired immune-deficiency syndrome':ab OR 'acquired immunedeficiency syndrome':ti OR 'acquired immunedeficiency syndrome':ab OR 'acquired immunodeficiency syndrome':ti OR 'acquired immunodeficiency syndrome':ab OR 'acquired immuno-deficiency syndrome':ti OR 'acquired immuno-deficiency syndrome':ab	568,176
#4	**#1 AND #2 AND #3**	835

References

1. Ford N, Shubber Z, Meintjes G, et al. Causes of hospital admission among people living with HIV worldwide: a systematic review and meta-analysis. *Lancet HIV* 2015; **2**(10): e438-44.
2. Wajanga BM, Webster LE, Peck RN, et al. Inpatient mortality of HIV-infected adults in sub-Saharan Africa and possible interventions: a mixed methods review. *BMC health services research* 2014; **14**: 627.
3. Akinkuotu A, Roemer E, Richardson A, et al. In-hospital mortality rates and HIV: A medical ward review, Lilongwe, Malawi. *International Journal of STD and AIDS* 2011; **22**(8): 465-70.
4. Myer L, Smith E, Mayosi BM. Medical inpatient mortality at Groote Schuur Hospital, Cape Town, South Africa, 2002-2009. *S Afr Med J* 2012; **103**(1): 28-31.
5. Hoffmann CJ, Milovanovic M, Cichowitz C, Kinghorn A, Martinson NA, Variava E. Readmission and death following hospitalization among people with HIV in South Africa. *PLoS ONE* 2019; **14**(7).
6. Laher AE, Paruk F, Venter W, Ayeni OA, Richards GA. Predictors of in-hospital mortality among HIV-positive patients presenting with an acute illness to the emergency department. *HIV Med* 2021.
7. Nemetchek B, English L, Kissoon N, et al. Paediatric postdischarge mortality in developing countries: A systematic review. *BMJ Open* 2018; **8**(12).
8. Long LC, Evans D, Rosen S, et al. Can routine inpatient mortality data improve HIV mortality estimates? Inpatient mortality at an urban hospital in South Africa. *S Afr Med J* 2018; **108**(10): 870-5.
9. Nijhawan AE, Clark C, Kaplan R, Moore B, Halm EA, Amarasingham R. An electronic medical record-based model to predict 30-day risk of readmission and death among HIV-infected inpatients. *Journal of Acquired Immune Deficiency Syndromes* 2012; **61**(3): 349-58.
10. Davy-Mendez T, Napravnik S, Wohl DA, et al. Hospitalization rates and outcomes among persons living with human immunodeficiency virus in the southeastern united states, 1996-2016. *Clinical Infectious Diseases* 2020; **71**(7): 1616-23.
11. Nijhawan AE, Kitchell E, Etherton SS, Duarte P, Halm EA, Jain MK. Half of 30-Day Hospital Readmissions among HIV-Infected Patients Are Potentially Preventable. *AIDS Patient Care and STDs* 2015; **29**(9): 465-73.
12. Gibson MP, Nijhawan AE, Jain MK, Halm E. Thirty-day readmissions among HIV-infected individuals at a safety-net hospital: Predictors and preventability. *Open Forum Infectious Diseases* 2016; **3**.
13. DerSimonian R, Laird N. Meta-analysis in clinical trials. *Control Clin Trials* 1986; **7**(3): 177-88.
